# Chlorophyll a gradients shift heterotrophic bacterial assembly from dispersal limitation to biotic filtering of picophytoplankton

**DOI:** 10.1093/ismeco/ycag215

**Published:** 2026-07-28

**Authors:** Pin-Yi Chen, Fuh-Kwo Shiah, Gwo-Ching Gong, Chih-hao Hsieh, Yi-Chun Yeh

**Affiliations:** Institute of Oceanography, National Taiwan University, Taipei, 10617, Taiwan; Research Center for Environmental Changes, Academia Sinica, Taipei, 11529, Taiwan; Institute of Marine Environment and Ecology, National Taiwan Ocean University, Keelung, 202-24, Taiwan; Center of Excellence for the Oceans, National Taiwan Ocean University, Keelung, 202-24, Taiwan; Institute of Oceanography, National Taiwan University, Taipei, 10617, Taiwan; Research Center for Environmental Changes, Academia Sinica, Taipei, 11529, Taiwan; Institute of Ecology and Evolutionary Biology, Department of Life Science, National Taiwan University, Taipei, 10617, Taiwan; National Center for Theoretical Sciences, Taipei, 10617, Taiwan; Institute of Oceanography, National Taiwan University, Taipei, 10617, Taiwan

**Keywords:** assembly process, heterotrophic bacterial community, chlorophyll a gradient, variation partitioning analysis, the southern East China Sea

## Abstract

The spatiotemporal dynamics of heterotrophic bacterial communities are critical for regulating elemental cycles. Yet, the processes governing the assembly of these communities across chlorophyll gradients remain unclear, especially regarding biotic interactions. Here, we present a framework that integrates biotic filtering of nanoplankton and picophytoplankton with environmental filtering and dispersal limitation to better elucidate the assembly processes of heterotrophic bacterial communities in a mesoscale marine environment. Microbial communities were collected along a 280 km east–west transect in the southern East China Sea during 13 research cruises conducted in late spring and summer during 2014–2018. Variation partitioning analysis explained 49.8%–78.1% of the variation in heterotrophic bacterial communities across cruises. As concentration of chlorophyll increased, dispersal limitation diminished, while biotic filtering of picophytoplankton intensified. Moreover, the ratio of deterministic to stochastic processes increased with chlorophyll a concentration. These results highlight deterministic processes, particularly biotic filtering of picophytoplankton, in structuring heterotrophic bacterial metacommunities during periods of high chlorophyll a concentration. Our findings emphasize the importance of incorporating biotic interactions into the framework of microbial community assembly to better understand microbial ecosystem responses to varying chlorophyll a conditions.

## Introduction

According to metacommunity theory, microbial communities are assembled through deterministic (niche-based) and stochastic (random) processes, or a combination of both [1–3]. Deterministic processes, including biotic filtering driven by species interactions and environmental filtering imposed by abiotic conditions, shape communities by favoring certain taxa. In contrast, stochastic processes, including dispersal limitation, involve random events that influence which taxa colonize and persist in a community. The relative influence of these processes is modulated by environmental conditions: highly selective environments amplify niche differences and promote deterministic assembly, while less selective environments increase the role of stochasticity [4]. While early studies focused on individual environmental parameters (eg, organic matter, pH, and nitrate) to explain soil bacterial community dynamics [4–6], studies of aquatic systems have since incorporated a broader suite of environmental variables to assess microbial assembly [7, 8]. However, in marine environments, the interplay between deterministic and stochastic processes across large spatial and temporal scales remains underexplored, particularly under selective conditions such as chlorophyll a concentrations, which can further alter community structure [9].

Chlorophyll a concentration, a widely used proxy of phytoplankton biomass and productivity, may influence microbial community assembly through phytoplankton-mediated biotic filtering. Elevated chlorophyll a concentrations are associated with increased phytoplankton-derived dissolved organic matter (DOM) [10], as well as changes in phytoplankton composition that alter the quantity and biochemical composition of released DOM [11]. Such shifts in the DOM composition alter niche conditions and selectively favor specific heterotrophic bacteria [12–14]. These phytoplankton-driven processes are key components of marine nutrient cycling, linking primary production to carbon transformation and the microbial loop and thereby influencing heterotrophic bacterial community structure and function. Although phytoplankton-associated changes in bacterial community assembly have been documented in freshwater [15–17] and brackish systems [18–20], the underlying mechanisms remain poorly resolved. In particular, it remains unclear whether elevated phytoplankton biomass alters bacterial assembly, for instance, by increasing labile resource availability, shifting phytoplankton community composition, or fundamentally altering the balance between deterministic and stochastic processes in marine ecosystems. Addressing these knowledge gaps is essential for understanding how bacterial communities respond to phytoplankton dynamics.

This study aims to evaluate the relative importance of environmental filtering, phytoplankton-mediated biotic filtering (including nanoplankton and picophytoplankton), and dispersal limitation in shaping heterotrophic bacterial metacommunities in the southern East China Sea (sECS) in response to varying chlorophyll a concentration. Using 16S and 18S rDNA amplicon sequencing in combination with variation partitioning analysis (VPA), we quantified the relative contributions of environmental, biotic, and spatial factors to heterotrophic bacterial community assembly along a repeated transect in the sECS. Regional chlorophyll a concentration was used as a proxy for overall phytoplankton biomass conditions. We hypothesized that: (i) elevated regional chlorophyll a conditions strengthen phytoplankton-mediated biotic control on heterotrophic bacterial communities through increased availability of phytoplankton-derived organic matter and enhanced ecological interactions; (ii) the relative importance of dispersal limitation decreases under high chlorophyll a conditions, as elevated chlorophyll a in this region is often associated with nutrient inputs from topographic upwelling or coastal eutrophic water masses that enhance water-mass connectivity and microbial exchange among stations [21–23]; (iii) environmental filtering remains an important driver across all conditions, although its relative contribution may vary with regional phytoplankton biomass; and (iv) bacterial community assembly shifts from being relatively more stochastic under low chlorophyll a conditions to more deterministic under elevated chlorophyll a conditions.

## Materials and methods

### Sampling sites and sample collection

**Figure 1:**
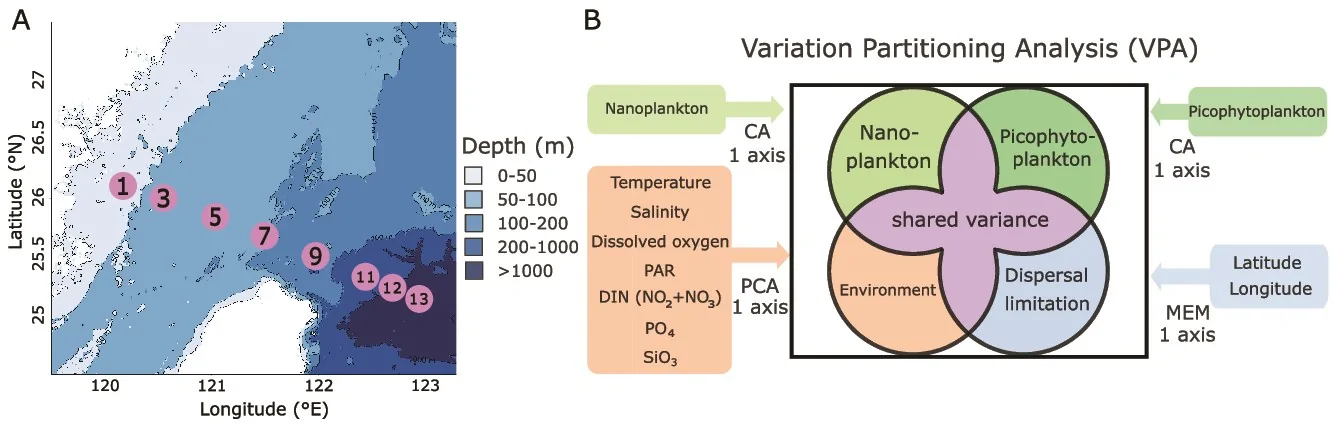
Sampling locations and workflow of variation partitioning analysis (VPA). (A) Sampling stations are indicated by numbers. Shelf regions are classified by bottom depth as inner shelf (0–50 m), middle shelf (50–100 m), outer shelf (100–200 m), and offshore (> 200 m). (B) Schematic overview of the analytical framework used to quantify heterotrophic bacterial assembly processes. Community composition, environmental variables, and spatial structure were first summarized using ordination methods, including correspondence analysis (CA), principal component analysis (PCA), and Moran’s eigenvector maps (MEMs). The best explanatory axis from each ordination was selected for the VPA to represent biotic filtering of nanoplankton and picophytoplankton, environmental filtering, and dispersal limitation. These four processes were subsequently partitioned to estimate their unique contributions to bacterial community assembly for each cruise.

This study involved a comprehensive sampling effort along the sECS transect (Fig. 1A). The transect included 8 stations spanning from coastal to oceanic waters along the continental shelf. From 2014 to 2018, a total of 13 cruises were conducted on the R/V *Ocean Researcher II*. Some stations in specific cruises were not sampled due to weather conditions (Table S1). At each station, two water layers were sampled: the surface layer (2–5 m) and the deep chlorophyll maximum (DCM) layer (8–90 m). The DCM depth was identified as the depth of peak chlorophyll fluorescence measured by a CTD profiler (SBE 9/11 plus CTD; Sea-Bird Electronics, Inc., USA) equipped with a fluorometer (AquaTracka III; Chelsea Technologies Group, Ltd., UK). In some cases, no DCM sample was collected because a distinct DCM was absent. Cruise information, sampling sites, and sampling depths are listed in Table S1. For clarity, each cruise was named after the sampling month and year (eg, cruise 7_2015).

A total of 144 seawater samples were collected by Go-Flo bottles (General Oceanics, Inc., USA) mounted on a rosette sampler. Twenty liters of seawater were collected from both surface and DCM layers and then prefiltered through a 20 μm mesh to remove larger organisms. The prefiltered seawater was then filtered through two size fractions (1.2 and 0.2 μm) using 142 mm polycarbonate membranes (Millipore, Inc., USA). All filtration was completed within 4 hours of sampling. Filters were flash-frozen in liquid nitrogen onboard and stored at −20°C until DNA extraction.

Seven environmental variables were measured at each sampling site. Temperature (°C), salinity (psu), and dissolved oxygen (mg/L) were measured with the CTD profiler. Seawater collected by Go-Flo bottles was used for nutrient analyses, including dissolved inorganic nitrogen (DIN, NO_2_ + NO_3_, μM), phosphate (μM), silicate (μM), and chlorophyll a (mg/m^3^) at each depth, following standard methods [23]. When nutrient data for the DCM layer were missing, linear interpolation was applied.

### DNA sequencing and sequence processing

DNA was extracted by the PowerWater DNA Extraction Kit (Qiagen, Inc., USA) following the manufacturer’s instructions. DNA from 1.2 μm pore-size filters was used for 18S rDNA amplicon sequencing [24] to target nanoplankton. DNA from the 0.2 μm pore-size filters was used for 16S rDNA amplicon sequencing [25] to target picophytoplankton and heterotrophic bacteria. A two-step PCR method was used [26] (see Supplementary method). PCR amplicons were sequenced on the Illumina MiSeq PE 300 platform (Illumina, Inc., USA). Sequences were processed into amplicon sequence variants (ASVs) using the DADA2 pipeline version 1.16 in R (v4.3.1) [27] and were deposited in the NCBI BioProject database under accession number PRJNA1393173. Taxonomic assignments for 18S ASVs were classified based on the Protist Ribosomal Reference (PR2) database (version 5.0) [28]. Taxonomic assignments for 16S ASVs were based on the Silva (version 138.1) [29] and the PhytoREF database [30]. To focus on unicellular protists, ASVs classified as Metazoa, Streptophyta, Fungi, and Opisthokonta were removed from the final 18S ASV table [31]. Contaminant sequences from mitochondria were also removed from the 16S ASV table. Rarefaction curves for both the 16S and 18S ASV datasets approached saturation across all samples, indicating that the sequencing depth was sufficient to capture most microbial diversity in the study area (Fig. S1). The 18S ASV table represented the nanoplankton community. The 16S ASV table was separated into picophytoplankton (cyanobacteria and eukaryotic phytoplankton) and heterotrophic bacterial communities (all bacteria excluding cyanobacteria).

### Community variation

The beta diversity of heterotrophic bacterial communities was illustrated by Principal Coordinates Analysis (PCoA) based on the Bray–Curtis distance. Variation of heterotrophic bacterial communities across chlorophyll a concentrations was examined by fitting a smooth surface of chlorophyll a onto the PCoA space with the ordisurf function from the R package vegan. Furthermore, dominant heterotrophic bacterial groups across chlorophyll a concentrations were identified by correlating their relative abundance with PCoA site scores using the envfit function from the R package vegan. Only order levels with a relative abundance greater than 1% and *P* values less than .05 were selected. When a taxon was unidentified at the order level, a higher taxonomic level was used. Nanoplankton and picophytoplankton communities were analyzed in the same manner to identify their dominant groups in relation to chlorophyll a concentrations.

### Regional chlorophyll a concentration

In this study, the cruise-averaged chlorophyll a concentration was calculated by averaging values across all sampling stations and depths (surface and DCM layers) to represent the regional chlorophyll a concentration for each sampled metacommunity (Fig. S2). Although this metric simplifies spatial heterogeneity along the transect, it effectively captures the macro-environmental conditions resulting from integrated hydrographic processes, including vertical mixing, upwelling, and water-mass variability. For example, lower regional chlorophyll a concentrations typically characterize oligotrophic conditions, in which phytoplankton biomass and phytoplankton-derived DOM are relatively low and growth is largely confined to the DCM layer. Conversely, higher regional chlorophyll a concentrations reflect enhanced phytoplankton biomass and phytoplankton-derived DOM. Ultimately, this proxy provides a framework for evaluating shifts in microbial communities across varying hydrographic regimes. Chlorophyll a variation (considering the surface and DCM layers) among sampling sites within each cruise was visualized using a bar plot, and differences among cruises were tested with a Kruskal–Wallis test followed by Dunn’s post hoc test. Additionally, to understand the environmental implications and vertical mixing effect on regional chlorophyll a concentration, cruise-averaged environmental parameters and mixed layer depth (MLD) were compared with regional chlorophyll a concentration using a linear model. The MLD was determined as the depth at which the temperature deviates from the 10 m temperature by 0.2°C [32].

### Assembly processes and their relationships with regional chlorophyll a concentration

We examined the relative contributions of various processes in influencing heterotrophic bacterial assembly, including biotic filtering of nanoplankton and picophytoplankton, environmental filtering, and dispersal limitation (Fig. 1B). For each cruise, the relative importance of these assembly processes was elucidated through variation partitioning analysis (VPA) [33], which decomposed beta diversity across sites for each transect, including data from both surface and DCM layers. First, heterotrophic bacterial communities were transformed into Bray–Curtis distance for each cruise. Second, Detrended Correspondence Analysis (DCA) was employed to determine the appropriate response model (Canonical Correspondence Analysis [CCA] or Redundancy Analysis [RDA]) for heterotrophic bacterial communities. Models were selected based on the longest gradient length derived from DCA results (CCA for ≥3; RDA for <3) [34]. In this study, RDA was suitable for all heterotrophic bacterial communities for all cruises. Therefore, partial distance-based RDA (partial dbRDA) was used in VPA.

To avoid overfitting, an improved two-step selection approach was used to incorporate each assembly process [35]. For biotic filtering, Correspondence Analysis (CA) was applied to the compositional data of nanoplankton. A stepwise forward selection method was used to select the most explanatory CA axis for the final VPA model. Likewise, biotic filtering of picophytoplankton was examined following the same analytical manner. For environmental filtering, Principal Component Analysis (PCA) was applied to the standardized environmental data, and the most explanatory PCA axis was selected. To assess dispersal limitation, Moran’s Eigenvector Maps (MEMs) [36] were calculated to characterize spatial variation and to analyze passive dispersal patterns structured by geographic distance, with the most explanatory MEM axis included in the final VPA model. Ultimately, the final VPA model incorporated 4 variables: biotic filtering of nanoplankton, biotic filtering of picophytoplankton, environmental filtering, and dispersal limitation. The uniquely explained variation for each variable was visualized in a bar plot. The ratio of deterministic to stochastic processes was estimated from the VPA results. The deterministic processes were defined as the sum of variation explained by biotic and environmental filtering. The effect of stochastic processes was defined as the community variation explained by dispersal limitation. In the last part, a linear model was used to determine whether the regional chlorophyll a concentration was related to the strengths of individual assembly processes across cruises. All statistical analyses and visualizations were conducted using R (v4.4.1).

## Results

### Variability in regional chlorophyll a concentration among cruises

Regional chlorophyll a concentration, quantified as cruise-averaged chlorophyll a concentration, ranged from 0.26 ± 0.15 to 1.75 ± 1.15 mg/m^3^ and differed significantly among cruises (Kruskal–Wallis test, *P* value <.001; Fig. S3). Pairwise comparisons revealed that the 3 cruises with the highest regional chlorophyll a concentration (cruise 7_2015, 5_2017, and 5_2016) were significantly different from the cruise with the lowest chlorophyll a concentration (cruise 5_2018; Dunn's post hoc test, *P* value <.05). Additionally, no clear seasonal pattern in regional chlorophyll a concentration was observed. Instead, regional chlorophyll a concentration was closely linked to nutrient availability and vertical mixing (Fig. S4). Cruise-averaged phosphate, DIN, and MLD were positively correlated with regional chlorophyll a concentration (phosphate versus regional chlorophyll a concentration: *R*^2^ = 0.433; *P* value = .015; DIN versus regional chlorophyll a concentration: *R*^2^ = 0.443; *P* value = .013; MLD versus regional chlorophyll a concentration: *R*^2^ = 0.532; *P* value = .005), while cruise-averaged silicate showed a marginally positive relationship (*R*^2^ = 0.243; *P* value = .087). Together, these results suggest that the regional chlorophyll a concentration at the mesoscale in the sECS was primarily regulated by nutrient concentrations and vertical mixing.

### Beta diversity of heterotrophic bacterial communities

**Figure 2:**
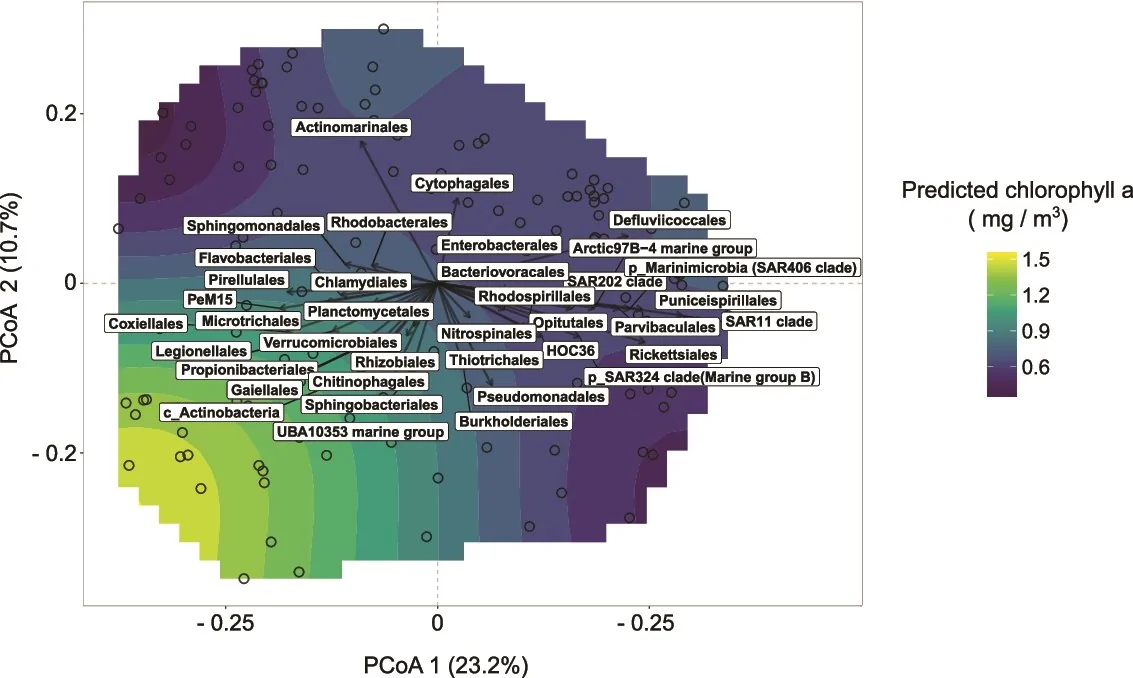
PCoA ordination of heterotrophic bacterial communities. Colors and smooth curves represent chlorophyll a concentrations. Vectors represent heterotrophic bacterial taxa significantly correlated with PCoA site scores (*P* value <.05). Heterotrophic bacteria are mainly labeled at the order level; those lacking order-level classification are labeled using higher-ranked taxa, with “p” denoting phylum and “c” denoting class.

In the PCoA space, distinct heterotrophic bacterial groups were distributed along the chlorophyll a gradient (Fig. 2). A total of 38 groups, mainly at the order level, were correlated with the PCoA axes. The top 10 groups included SAR11 clade (correlation coefficient, 0.91), Rickettsiales (0.7), Puniceispirillales (0.64), Defluviicoccales (0.58), phylum_Marinimicrobia (SAR406 clade) (0.56), Parvibaculales (0.5), Actinomarinales (0.39), PeM15 (uncultured Actinobacteria; 0.38), Coxiellales (0.38), and Arctic97B-4 marine group (0.38). The community structure was primarily shaped by oligotrophic bacteria, such as SAR11 and Puniceispirillales, which were associated with lower chlorophyll a concentrations. In contrast, phytoplankton-associated bacteria, including Flavobacteriales and Rhodobacterales, showed lower correlation coefficients (0.14 and 0.07, respectively), and were more prevalent at higher chlorophyll a levels. These results indicate that environments with different chlorophyll a concentrations support distinct functional groups of heterotrophic bacteria.

### Beta diversity of picophytoplankton and nanoplankton communities

Different picophytoplankton groups, mainly at the order level, dominated across the chlorophyll a gradient (Fig. S5). In the PCoA of picophytoplankton communities, the first two axes explained 62.1% of the variation. Among the dominant groups, 10 groups were correlated with the PCoA axes; these were Synechococcales (correlation coefficient, 0.7), Pelagomonadales (0.65), Mamiellales (0.6), Prymnesiophyceae (0.55), Prymnesiales (0.48), Phaeocystales (0.43), Pyrenomonadales (0.14), Prasino-clade-7 (0.13), Trebouxiophyceae (0.1), and Chrysophyceae-Synurophyceae (0.05). Synechococcales were primarily associated with high chlorophyll a levels, whereas eukaryotic picophytoplankton were more abundant under lower chlorophyll a levels. Two groups of unicellular green algae, Prasino-clade-7 and Trebouxiophyceae, also showed significantly positive associations with high chlorophyll a, although their correlation coefficients were comparatively weak.

In the PCoA of nanoplankton communities, the first 2 axes explained 20.7% of the variation (Fig. S6). In total, 49 groups, mainly at the order level, were correlated with the PCoA axes. The 10 most dominant groups were Chlorellales (correlation coefficient, 0.6), Dino-Group-I (0.45), Pelagomonadales (0.43), Prorocentrales (0.42), Telonemia (0.38), Dino-Group-III (0.37), Prasino-Clade-9 (0.36), Gymnodiniales (0.36), Nanomonadea (0.35), and Bacillariales (0.3). Chlorellales, Chloropicales, and Prasinodermales, identified as phototrophic plankton [31], were associated with high chlorophyll a levels. In contrast, Dino-Group-I, Pelagomonadales, and Prorocentrales were more prevalent in low chlorophyll a environments. Nanoplankton and picophytoplankton associated with high chlorophyll a levels may play a role in biotic filtering effects on heterotrophic bacterial communities.

### Assembly processes of heterotrophic bacterial communities in relation to regional chlorophyll a concentration

**Figure 3:**
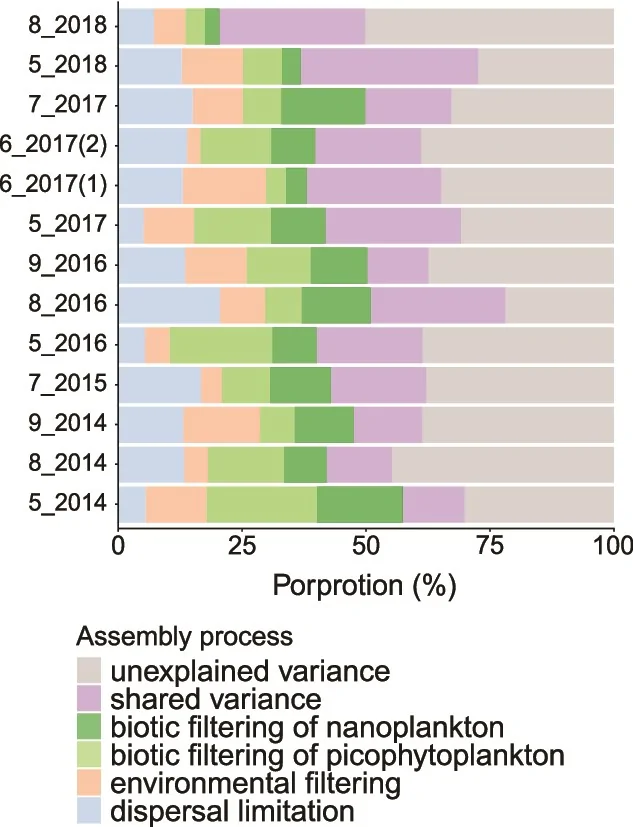
Variation partitioning results for heterotrophic bacterial metacommunities across cruises.

The VPA explained 49.8%–78.1% of the variation in heterotrophic bacterial metacommunities across cruises (Fig. 3). After accounting for shared variation among factors (12.4%–35.8%), the unique contributions of individual processes were quantified. Dispersal limitation explained 5.1%–20.5% of the variation, environmental filtering accounted for 2.7%–16.8%, biotic filtering of picophytoplankton contributed 3.9%–22.3%, and biotic filtering of nanoplankton explained 2.9%–17.3%. On average, dispersal limitation represented the largest unique fraction (mean = 11.9%), followed by biotic filtering of picophytoplankton (11.4%), biotic filtering of nanoplankton (10.1%), and environmental filtering (9.4%). Although the mean contributions of these assembly processes were similar, their relative importance varied among cruises.

**Figure 4:**
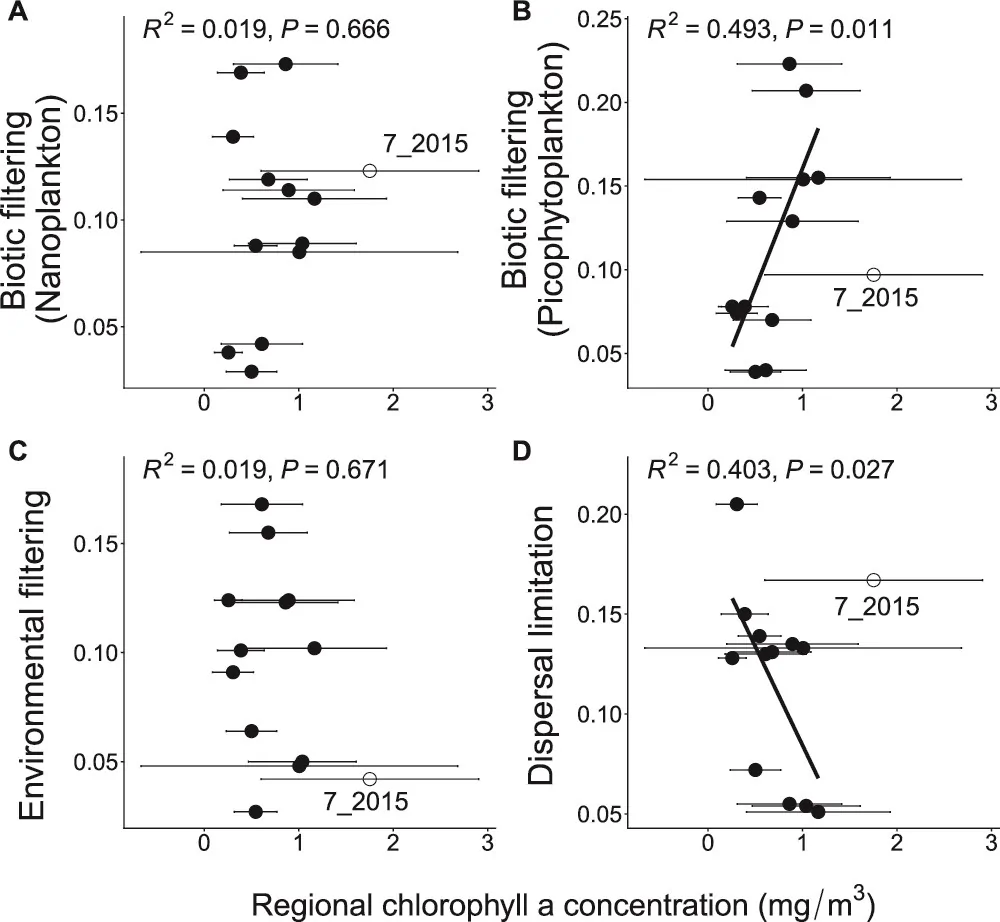
Relationship between chlorophyll a concentration and each analyzed assembly process, excluding cruise 7_2015 (represented by an open circle). The black line represents the significant linear relationship fitted using linear models. Horizontal bars represent the standard deviation of chlorophyll a concentration for each cruise.

**Figure 5:**
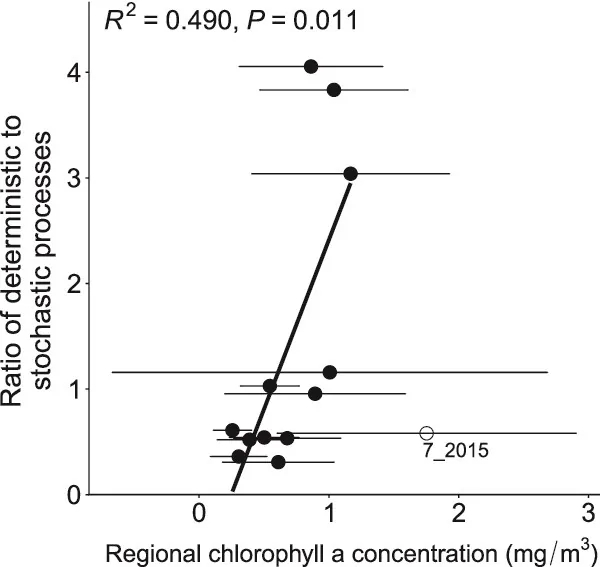
Relationship between chlorophyll a concentration and the ratio of deterministic to stochastic processes, excluding cruise 7_2015 (represented by an open circle). The black line represents the significant linear relationship fitted using a linear model. Horizontal bars represent the standard deviation of chlorophyll a concentration for each cruise.

No significant relationships were observed between community assembly processes and regional chlorophyll a concentration when all cruises were included, largely due to the influence of a single high regional chlorophyll a concentration outlier (cruise 7_2015; Fig. S7). After excluding cruise 7_2015 (see Discussion for justification) and reanalyzing the data, several significant associations emerged between assembly processes and regional chlorophyll a concentration (Fig. 4). Specifically, biotic filtering of picophytoplankton was positively correlated with regional chlorophyll a concentration (Fig. 4B; *R*^2^ = 0.493; *P* value = .011), while dispersal limitation showed a negative correlation (Fig. 4D; *R*^2^ = 0.403; *P* value = .027). However, environmental filtering (Fig. 4C; *P* value = .671) and biotic filtering of nanoplankton (Fig. 4A; *P* value = .666) remained uncorrelated with regional chlorophyll a concentration, even after excluding cruise 7_2015. When assembly processes were further grouped into niche-based and neutral-based components, the ratio of deterministic to stochastic effects was positively correlated with regional chlorophyll a concentration (Fig. 5; *R*^2^ = 0.49; *P* value = .011).

## Discussion

### Increased biotic filtering of picophytoplankton with regional chlorophyll a concentration

The strength of biotic filtering exerted by picophytoplankton on heterotrophic bacterioplankton increased with regional chlorophyll a concentration. This finding aligns with previous observations by Wang et al. [15], which reported that picophytoplankton can alter heterotrophic bacterial communities under elevated chlorophyll a conditions. Although VPA identifies the statistical contributions of different factors to community variation, these relationships remain correlative and do not inherently demonstrate direct causal interactions. Nevertheless, recent experimental evidence supports the plausibility of such interactions by showing that picophytoplankton, such as *Synechococcus,* actively engage in metabolic interactions with heterotrophic bacteria by releasing nitrogen-rich DOM, which can be efficiently exploited by chemotactic bacteria [37]. Although direct evidence linking Synechococcales-derived DOM to bacterial community shifts in the sECS is still lacking, to our knowledge our results provide the first empirical indication that picophytoplankton composition may play a significant role in shaping heterotrophic bacterial communities during high chlorophyll a concentration in a marine ecosystem. Worth noting, our findings do not suggest that picophytoplankton exert a greater impact than larger phytoplankton, which are traditionally considered the dominant primary producers on continental shelves [38–40]. Rather, we highlight that the role of picophytoplankton in this system has often been overlooked. In fact, picophytoplankton frequently coexist with larger phytoplankton species [41] and can exhibit significantly elevated productivity during periods of high chlorophyll a concentration [42, 43]. Our findings suggest that this small-sized fraction of the autotrophic community represents a crucial, yet underappreciated, driver of microbial community assembly.

Conversely, the biotic filtering of nanoplankton on heterotrophic bacteria showed no relationship with chlorophyll a concentration. Several reasons may account for this pattern. First, nanoplankton predation on bacterial community structure was relatively weak from a community assembly perspective. Phagotrophic nanoplankton, such as ciliates and nanoflagellates, are major bacterivores that facilitate energy transfer within the microbial food web and can influence bacterial composition and abundance through bacterivory [44–46]. In this study, however, nanoplankton composition was not correlated with bacterial communities, suggesting that the effect of nanoplankton on bacteria is primarily abundance dependent rather than species dependent [35, 47]. Second, an increasing number of nanoplankton taxa are recognized as mixotrophs capable of both phototrophy and phagotrophy [48]. For example, Chlorellales, the dominant nanoplankton associated with high chlorophyll a concentration in this study, may function as either phototrophic or mixotrophic organisms [49] (Fig. S6). Yet, few studies have examined how mixotrophic nanoplankton influence bacterial communities [50]. Such dual nutritional strategies complicate assessments of biotic filtering, especially when nanoplankton composition varies across chlorophyll a gradients. Despite the strengthening biotic influence of picophytoplankton on heterotrophic bacteria with increasing regional chlorophyll a concentration, the ecological role of nanoplankton in shaping bacterial dynamics remains unresolved and warrants further investigation.

### Decreased dispersal limitation with regional chlorophyll a concentration

To our knowledge, we report the reduced dispersal limitation of heterotrophic bacterial communities with increasing regional chlorophyll a concentration for the first time at the mesoscale. Under low regional chlorophyll a concentration, the separation of inshore and offshore water masses creates physical barriers for heterotrophic bacterial metacommunities, a phenomenon often observed in coastal systems [51]. However, the T–S diagram indicates that the entire sECS was largely homogenized by the Kuroshio Current (KC) or Taiwan Strait Current (TSC) in late spring and summer (Fig. S8). This finding suggests that smaller-scale currents, such as intra-diurnal tidal currents [52, 53] or mesoscale countercurrents [54, 55], may create physical barriers that are not resolved in T–S diagrams and thus may have been overlooked. As regional chlorophyll a concentration increases, strong upwelling or river discharge weakens the inshore–offshore spatial structure for the sECS, resulting in reduced dispersal limitation. Overall, higher regional chlorophyll a concentrations correspond to weaker dispersal limitation of heterotrophic bacteria across broad spatial scales.

In contrast to other cruises, cruise 7_2015 represents an exception to the general pattern, showing strong dispersal limitation despite high regional chlorophyll a concentration. During this cruise, spatial structuring of heterotrophic bacterial communities was more strongly associated with the dispersal limitation variable (MEM 1) than with other MEM variables (Fig. S9), indicating the presence of pronounced physical barriers. Two mechanisms may explain this exception. First, a 30-m thick freshwater pulse was detected at the surface of station 7 (Fig. S9), likely resulting from intense summer monsoon rainfall and river discharge from western Taiwan (Fig. S10). A similar freshwater pulse driven by a typhoon has been reported previously in northwest Taiwan [56]. This brackish water influx likely introduced terrestrial nutrients that fueled the high chlorophyll a concentration. Additionally, the strong summer monsoon may have intensified the TSC, reinforcing the physical boundary between the TSC and KC (Fig. S9). This interpretation matches earlier observations that bacterial communities in different water masses become more distinct during high productivity periods [57]. Thus, these monsoon-driven processes likely explain the atypical coexistence of high regional chlorophyll a concentration and strong dispersal limitation observed using cruise 7_2015, representing an exception to the hypothesized chlorophyll a–dispersal relationship.

### Environmental filtering in chlorophyll a gradients

Environmental filtering exhibited no relationship with regional chlorophyll a concentration in heterotrophic bacteria. The average proportion of variation explained by environmental filtering was also lower than biotic filtering. Previous studies have also found that environmental effects are often less influential than biotic interactions in shaping bacterial communities [20, 58]. One explanation is ecological buffering, in which interactions among organisms reduce their sensitivity to environmental fluctuations [15, 59]. Another possibility is that the spatial scale of this study may not have been large enough to capture strong environmental gradients, as environmental filtering tends to be more apparent at broader spatial scales [60]. For instance, in the Baltic Sea, salinity and temperature strongly influence bacterial community assembly [20]. In contrast, the sECS environment is often homogenized by dominant water masses (Fig. S8), which may explain the relatively weak effect of environmental filtering observed in this study. These findings underscore the importance of considering both spatial scale and biotic interactions when analyzing bacterial community assembly processes.

### Increased ratio of deterministic to stochastic effects with regional chlorophyll a concentration

The balance between deterministic and stochastic assembly processes shifted systematically along the gradient of regional chlorophyll a concentration. With sufficient nutrients, the environment not only promotes higher regional chlorophyll a concentration but also favors more deterministic assembly processes in heterotrophic bacterial metacommunities relative to stochastic processes. Similar patterns have been reported in previous studies, where deterministic processes dominate during elevated chlorophyll conditions [16, 19], although exceptions exist [17, 61]. These observations suggest that the balance between deterministic and stochastic influences during high chlorophyll is not fixed but varies dynamically with environmental context and dominate phytoplankton taxa. Collectively, these results indicate that changes in chlorophyll a can fundamentally reshape the mechanisms governing heterotrophic bacterial community assembly.

### Heterogeneous chlorophyll a patterns in the sECS

Cruise-averaged chlorophyll a concentration, used here as a proxy for regional chlorophyll a concentration, may not fully resolve the spatial and temporal heterogeneity of the sECS. Owing to the strong spatial variability of chlorophyll a distributions driven by complex hydrographic conditions [21, 22], similar regional chlorophyll a concentrations can arise from different spatial distributions. In this study, high regional chlorophyll a concentration resulted from coastal (eg, cruise 8_2014), offshore (eg, cruise 5_2017), or northwestern Taiwan (eg, cruise 7_2015) (Fig. S2). Despite this heterogeneity, the phytoplankton-associated effects on heterotrophic bacterial metacommunities are likely to extend beyond local high chlorophyll a patches through regional dispersal among sites. Accordingly, our results suggest that chlorophyll a concentration, when interpreted at the cruise scale, serves as a useful proxy for capturing regional changes in bacterial community assembly processes in the sECS.

## Conclusion

By integrating biotic and abiotic parameters with multi-year data from a marine ecosystem, this study examined changes in the assembly processes of heterotrophic bacterial communities. Our results show that, at the mesoscale, increasing regional chlorophyll a concentration is associated with stronger biotic filtering of picophytoplankton and reduced dispersal limitation. Furthermore, the relative importance of deterministic processes compared to stochastic processes increases with regional chlorophyll a concentration, aligning with previous studies that report stronger deterministic influences during high chlorophyll conditions [15, 18]. Among deterministic processes, biotic filtering has a greater effect than environmental filtering, likely due to ecological buffering, where strong biotic interactions reduce sensitivity to environmental variability. These findings highlight the dynamic change of heterotrophic bacterial community assembly across varying chlorophyll a.

## Supplementary Material

0720_ISMEcomm_supp_Final_ycag215

## Data Availability

The code underlying this study is available at doi: 10.5281/zenodo.18396706.
