# Supplementary material for: Chlorophyll a gradients shift heterotrophic bacterial assembly from dispersal limitation to biotic filtering of picophytoplankton
Source: ISME Commun. 2026 Jul 28;6(1):ycag215. doi: 10.1093/ismeco/ycag215 (PMC13561414; doi:10.1093/ismeco/ycag215)
Supplement: Supplementary file 1 [file 0720_ismecomm_supp_final_ycag215.pdf]

Supplementary information

Chlorophyll a gradients shift heterotrophic bacterial assembly from dispersal limitation to biotic filtering of picophytoplankton

Short title: **Chlorophyll a gradients shift heterotrophic bacterial assembly**

Pin-Yi Chen[1], Fuh-Kwo Shiah[2], Gwo-Ching Gong[3,4], Chih-hao Hsieh[1, 2, 5, 6] and Yi-Chun Yeh[1*]

[1]Institute of Oceanography, National Taiwan University, Taipei, Taiwan

[2]Research Center for Environmental Changes, Academia Sinica, Taipei, Taiwan

[3]Institute of Marine Environment and Ecology, National Taiwan Ocean University, Keelung, Taiwan

[4]Center of Excellence for the Oceans, National Taiwan Ocean University, Keelung, Taiwan

[5]Institute of Ecology and Evolutionary Biology, Department of Life Science, National Taiwan University, Taipei, Taiwan

[6]National Center for Theoretical Sciences, Taipei, Taiwan

Methods

A two-step PCR method was followed by Berry et al. [1]. The 18S and 16S rDNA were separately amplified. The V4 region (~270 bp) of the 18S rDNA was targeted using the forward primer TAReuk454FWD1 (5'-[forward index adaptor]-CCAGCA(G/C)C(C/T)GCGGTAATTCC-3') and reverse primer TAReukREV3 (5'-[reverse index adaptor]-ACTTTCGTTCTTGAT(C/T)(A/G)A-3) [2]. The V4–V5 region (~400 bp) of the 16S rDNA was amplified using the forward primer 518F (5'-[forward index adaptor]- CCAGCAGCYGCGGTAAN -3') and reverse primer 926R (5'-[reverse index adaptor]- CCGTCAATTCNTTTRAGT, CCGTCAATTTCTTTGAGT and CCGTCTATTCCTTTGANT -3') which followed Visualization and Analysis of Microbial Population Structures (VAMPS) website (https://vamps2.mbl.edu/) [3].

References

1. Berry D, Ben Mahfoudh K, Wagner M, Loy A. Barcoded primers used in multiplex amplicon pyrosequencing bias amplification. *Appl Environ Microbiol* 2011;**77**:7846-9. https://doi.org/10.1128/AEM.05220-11
2. Stoeck T, Bass D, Nebel M, Christen R, Jones MDM, Breiner HW, et al. Multiple marker parallel tag environmental DNA sequencing reveals a highly complex eukaryotic community in marine anoxic water. *Mol Ecol* 2010;**19**:21-31. https://doi.org/10.1111/j.1365-294X.2009.04480.x
3. Huse SM, Welch DBM, Voorhis A, Shipunova A, Morrison HG, Eren AM, et al. VAMPS: a website for visualization and analysis of microbial population structures. *BMC Bioinformatics* 2014;**15**:41. https://doi.org/10.1186/1471-2105-15-41

Tables

Table S1. Cruise information, sampling stations, and sampling depths. DCM indicates deep chlorophyll maximum. The empty cells arise from a lack of sampling due to weather conditions or the absence of a distinct DCM.

Cruise	Sample name	Time	Layer	Station / Sampling depth (m)							
				1	3	5	7	9	11	12	13
Cr2023	5_2014	2014/5/25-5/29	Surface		3	3	3	3			
			DCM		20	20			10		80
Cr2046	8_2014	2014/8/13-8/15	Surface	3	3	3	3	3	3	3	
			DCM		25		45	30	40	20	
Cr2053	9_2014	2014/9/9-9/11	Surface	3	3	3	3	3	3	3	
			DCM		30	25	25	50	30	50	
Cr2109	7_2015	2015/7/20-7/24	Surface	3	3	3	3	3			3
			DCM	10	20	20	8	60			70
Cr2164	5_2016	2016/5/10-5/13	Surface		3	3	3	3	3		
			DCM	12	18	10		18	20		
Cr2183	8_2016	2016/8/8-8/10	Surface		3	3	3	3		3	
			DCM		25	35		35			
Cr2192	9_2016	2016/9/1-9/2	Surface	3	3	3	3	3	3		
			DCM		10	27	25		20	50	
Cr2241	5_2017	2017/5/26-5/29	Surface	5	5	5	5	5	5	5	
			DCM		25	30	10			60	

Table S1. Continued.

Cruise	Sample name	Time	Layer	Station / Sampling depth (m)							
				1	3	5	7	9	11	12	13
Cr2243	6_2017(1)	2017/6/6-6/9	Surface		3	3	3	3	3	3	
			DCM		20	40	20	45	13	90	
Cr2245	6_2017(2)	2017/6/18-6/20	Surface	3	3	3	3	3	3	3	
			DCM	20	30	15			40	10	
Cr2248	7_2017	2017/7/1-7/4	Surface		3	3	3	3	3		
			DCM		25	35	35	47	65	60	
Cr2304	5_2018	2018/5/17-5/19	Surface	3	3	3	3			3	
			DCM	15	30	30	35			55	
Cr2324	8_2018	2018/8/1-8/4	Surface	5	5	5	5	5	5	5	
			DCM	17	24	25	45	50	30	35	

Figure Legends

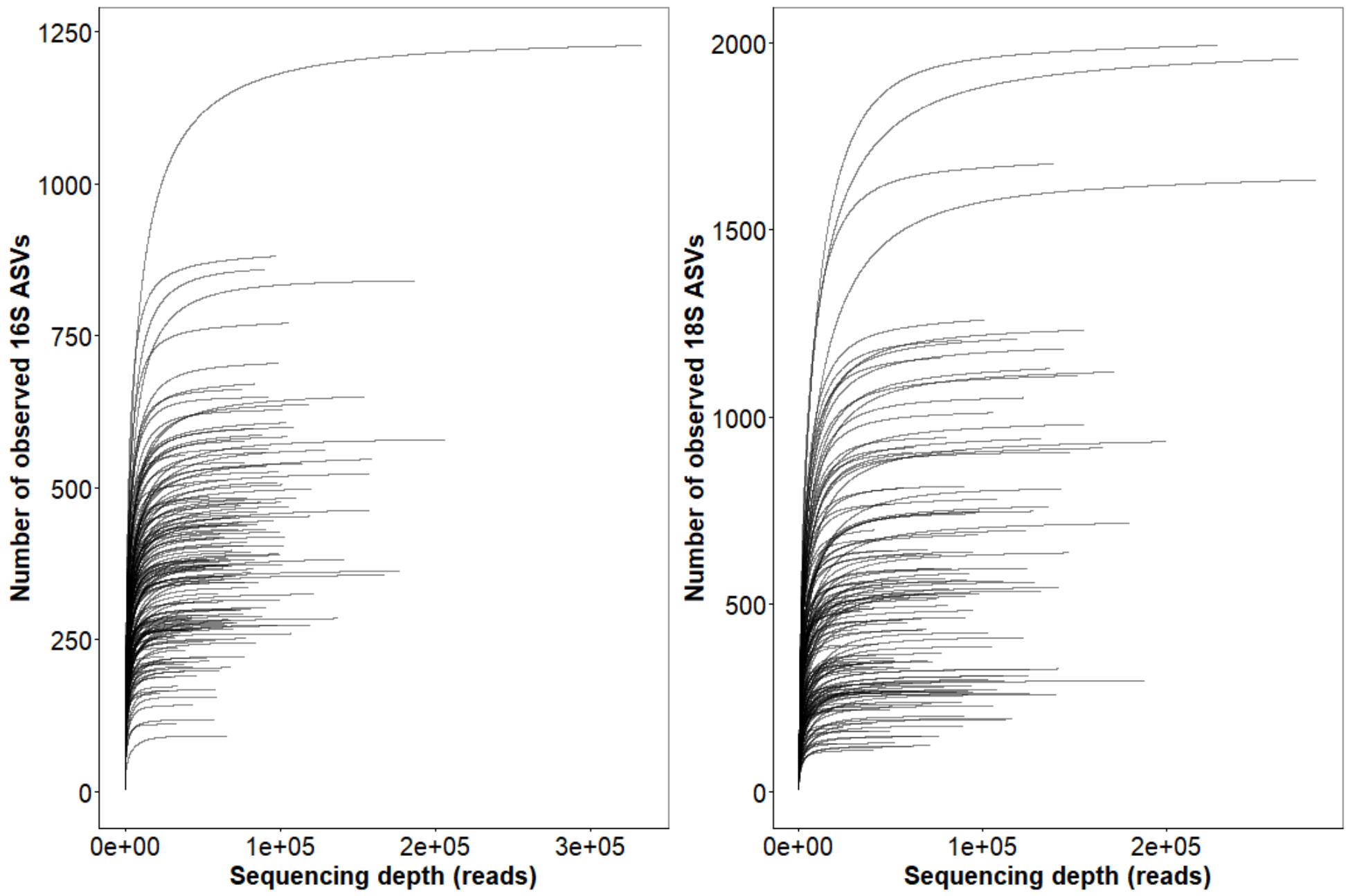

Figure S1. Rarefaction curves of 16S and 18S rDNA amplicon sequencing. Most curves approach an asymptotic plateau, demonstrating that the current sequencing depth provides a representative coverage of the microbial communities.

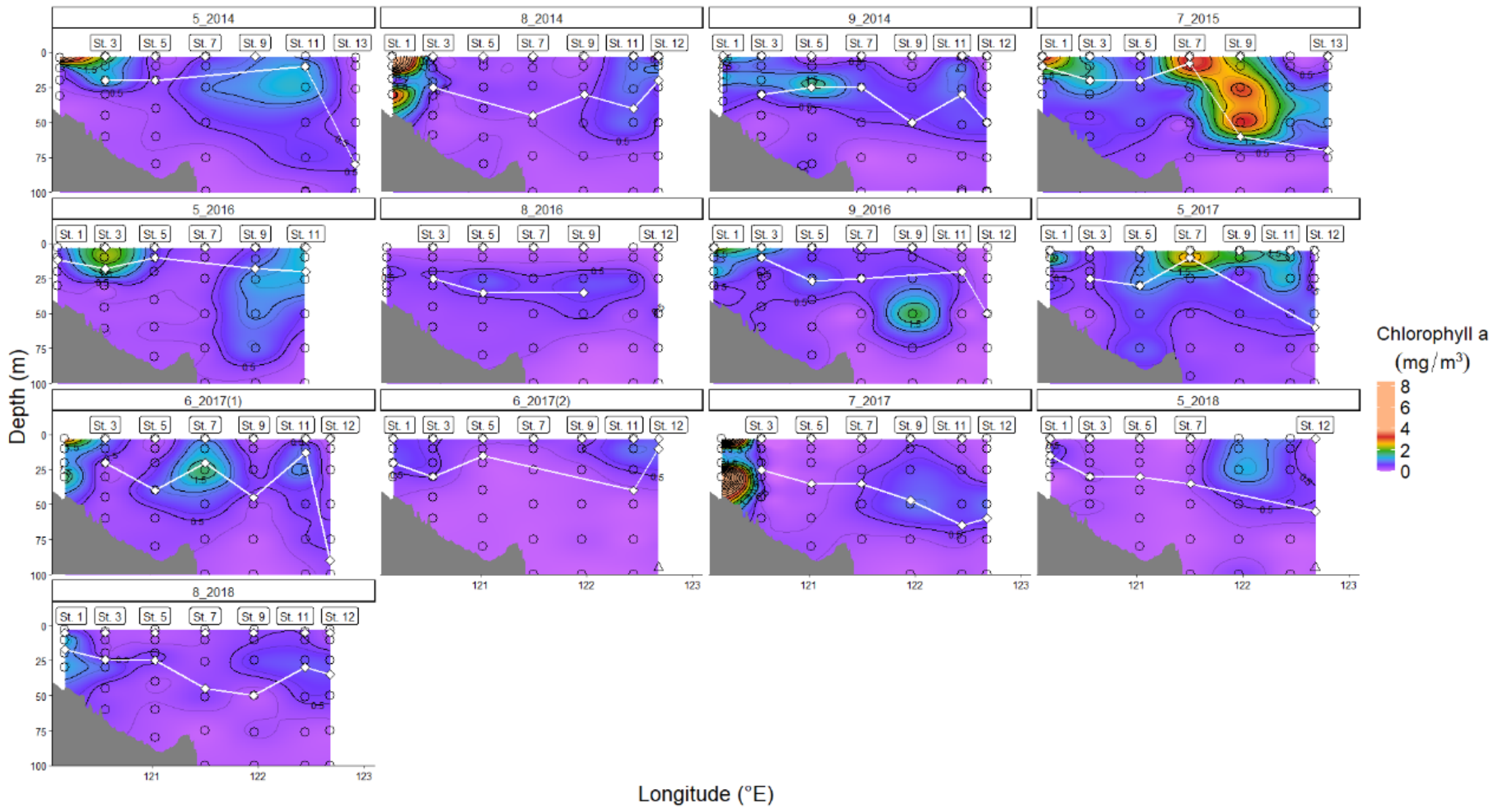

Figure S2. Vertical profiles of chlorophyll a for each cruise. Each diamond represents bacterial community sampling depths, and hollow circles represent nutrient sampling depths. Some missing diamonds arise from a lack of sampling due to weather conditions or the absence of a distinct DCM. The white line represents the possible distribution of the deep chlorophyll maximum (DCM) layer. Thick and thin black lines represent 0.5 and 0.3 (mg/m^3) contour intervals, respectively.

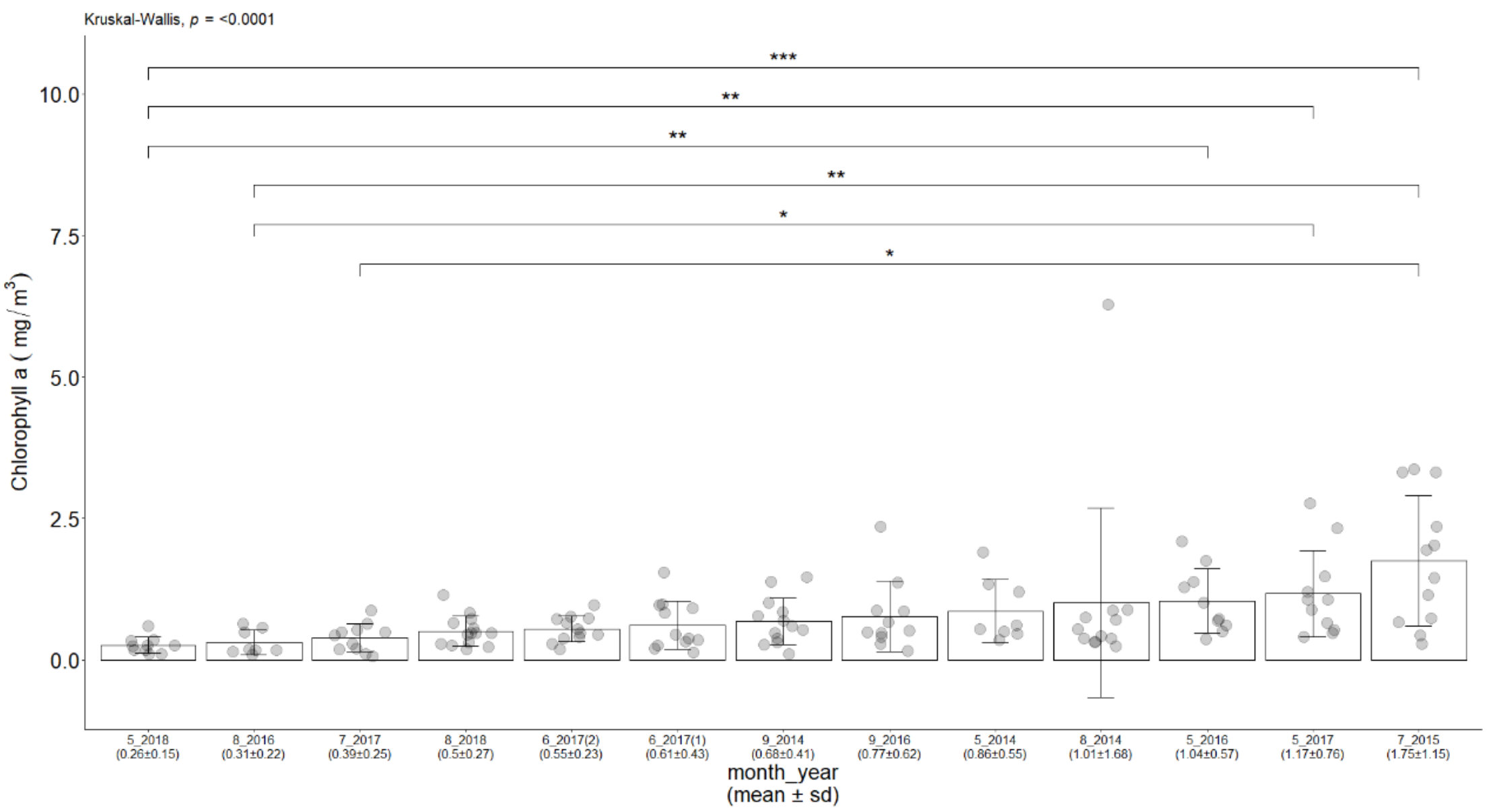

Figure S3. Bar plot of Kruskal-Wallis test and Dunn post-hoc test for comparing chlorophyll a concentrations across cruises. Each dot represents an individual chlorophyll a datum for each sample. Bars represent cruise-averaged chlorophyll a, indicating regional chlorophyll a concentration. Error bars represent the standard deviation within each cruise. Significance levels for Dunn post-hoc test: *** for p-value < 0.001, ** for p-value < 0.01, * for p-value < 0.05.

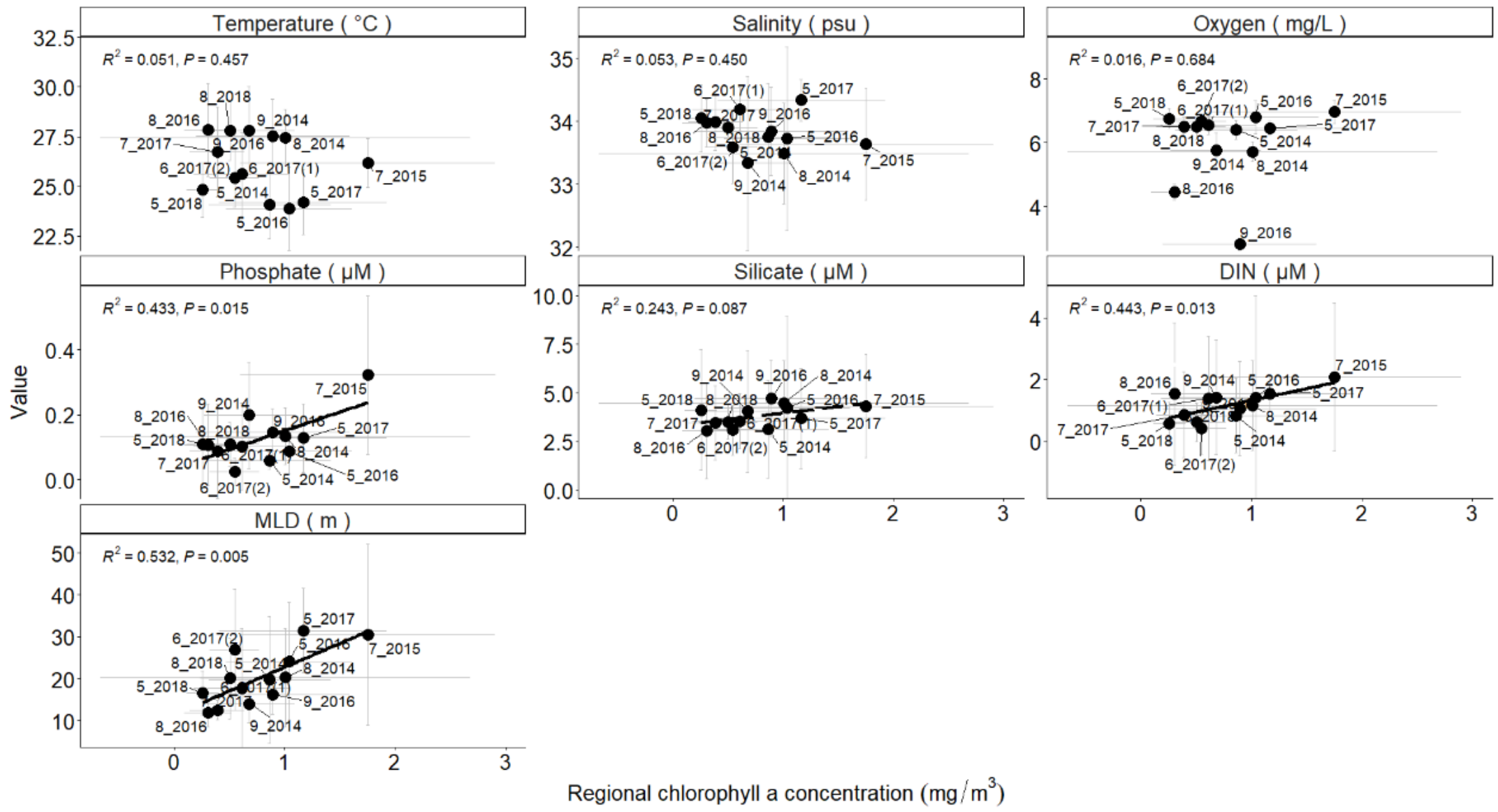

Figure S4. Relationships between regional chlorophyll a concentration and environmental variables across cruises. The black line represents the significant linear relationship, while the dashed line represents a marginally significant relationship through linear regression. Error bars show the standard deviation within each cruise. MLD represents mixed layer depth.

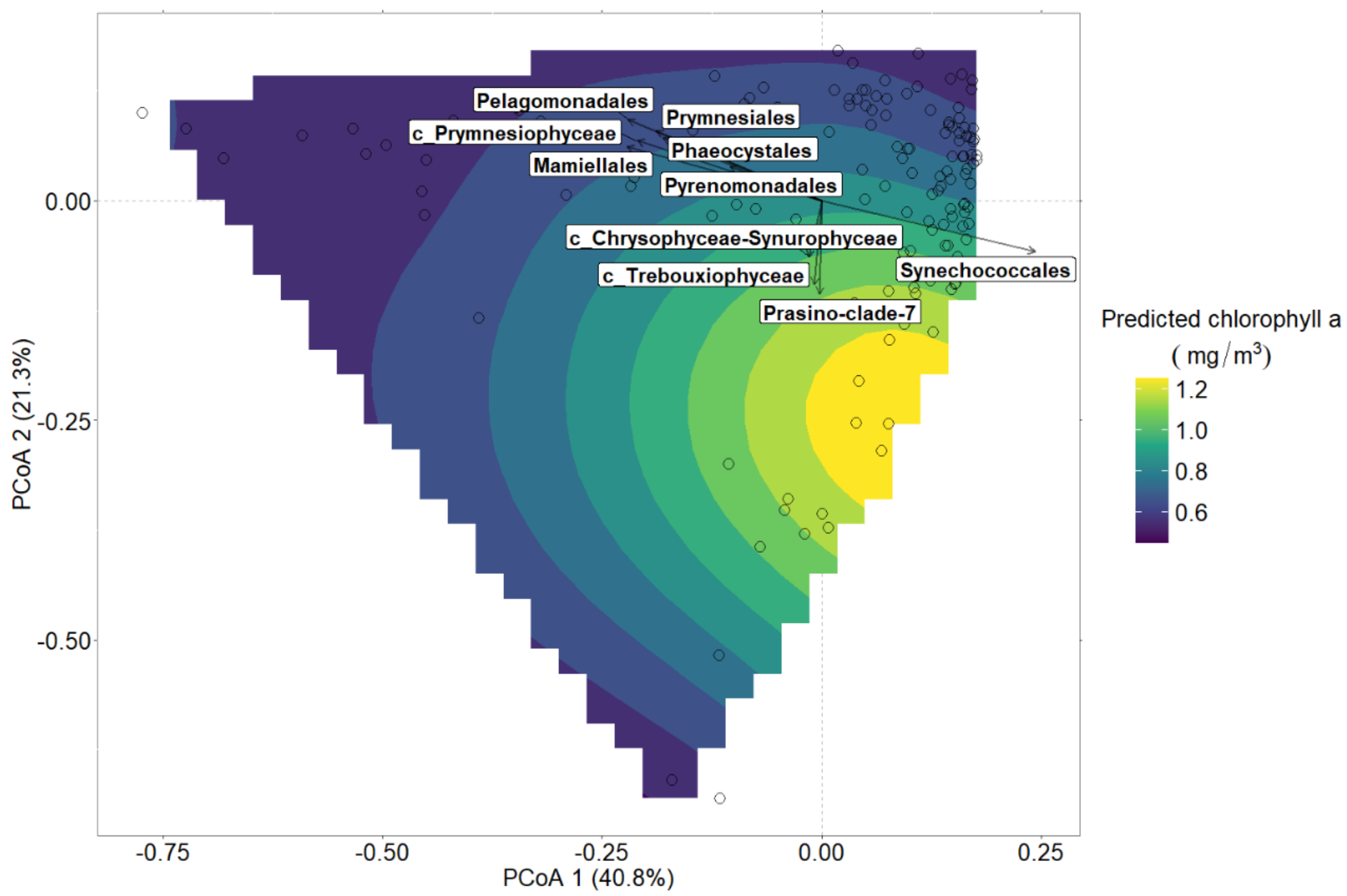

Figure S5. PCoA plot of picophytoplankton communities. Colors and smooth curves represent chlorophyll a. Each vector represents a picophytoplankton group significantly correlated with PCoA site scores (p-value < 0.05). Missing order names are replaced with higher-ranked taxa: “c” for class rank.

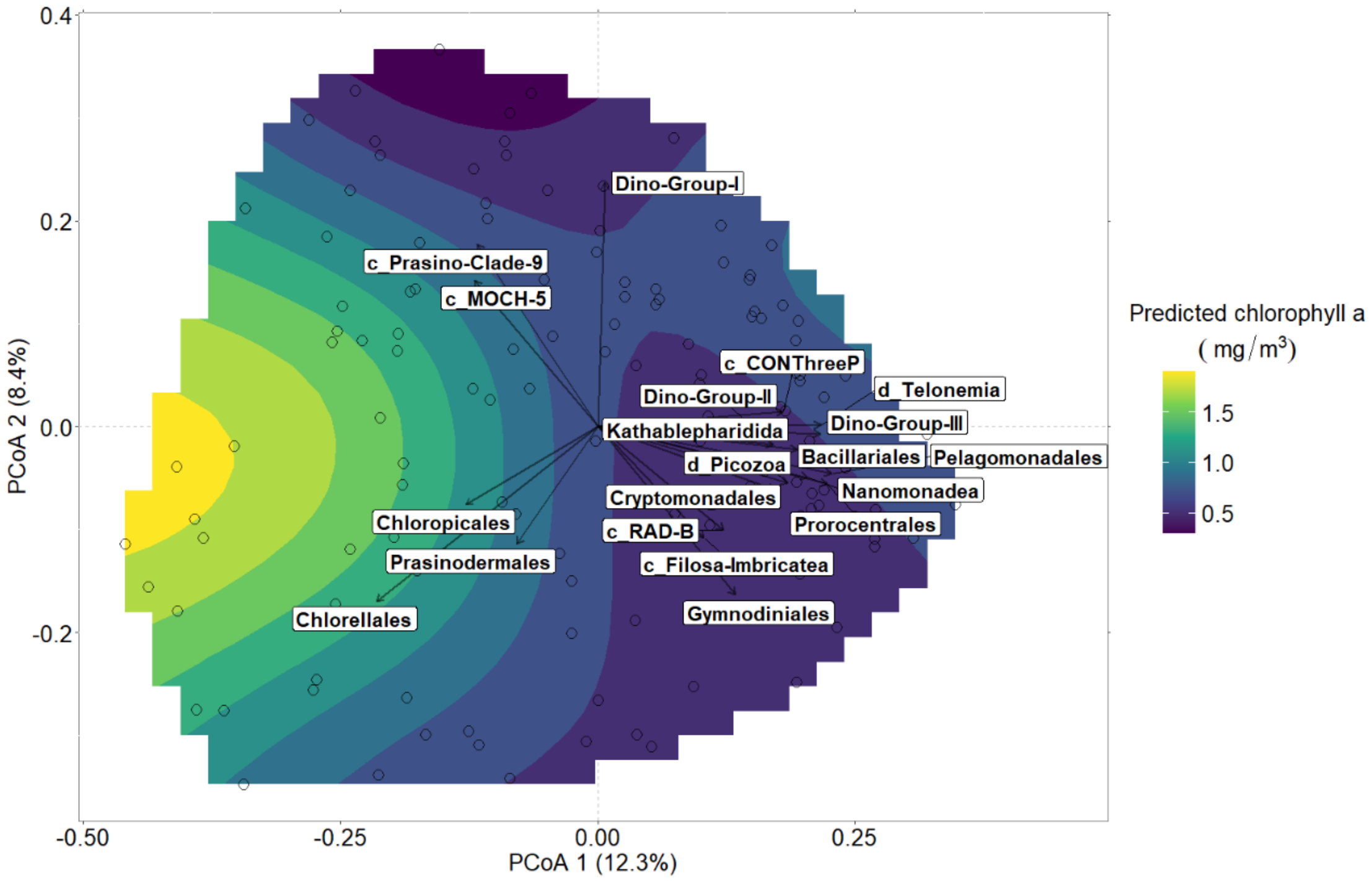

Figure S6. PCoA plot of nanoplankton communities. Colors and smooth curves represent chlorophyll a. Each vector represents a nanoplankton group significantly correlated with PCoA site scores (p-value < 0.05). Missing order names are replaced with higher-ranked taxa: “d” for division rank, “c” for class rank.

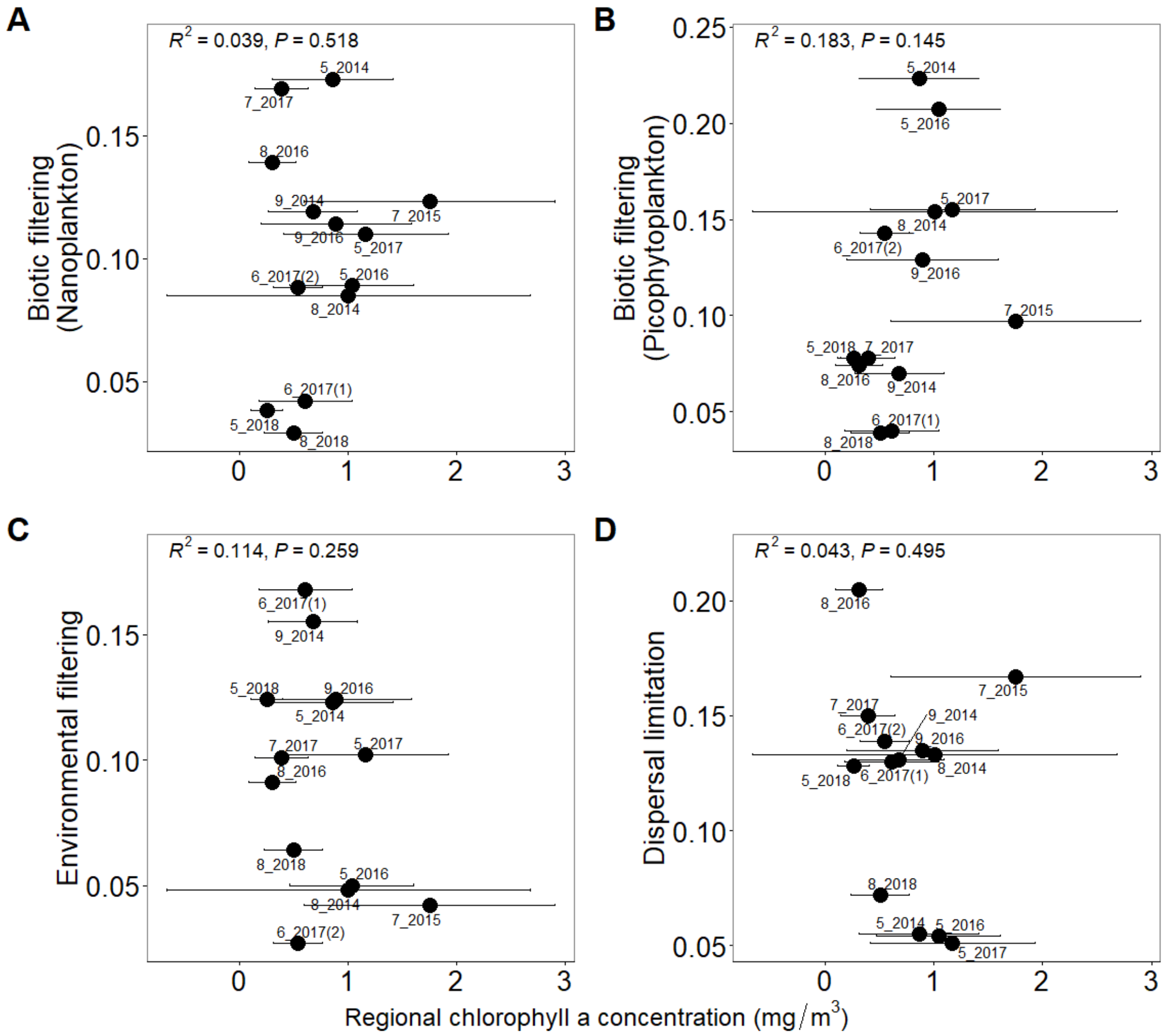

Figure S7. Relationship between regional chlorophyll a concentration and each of the analyzed assembly processes. The horizontal bar represents the standard deviation of chlorophyll *a* in each cruise.

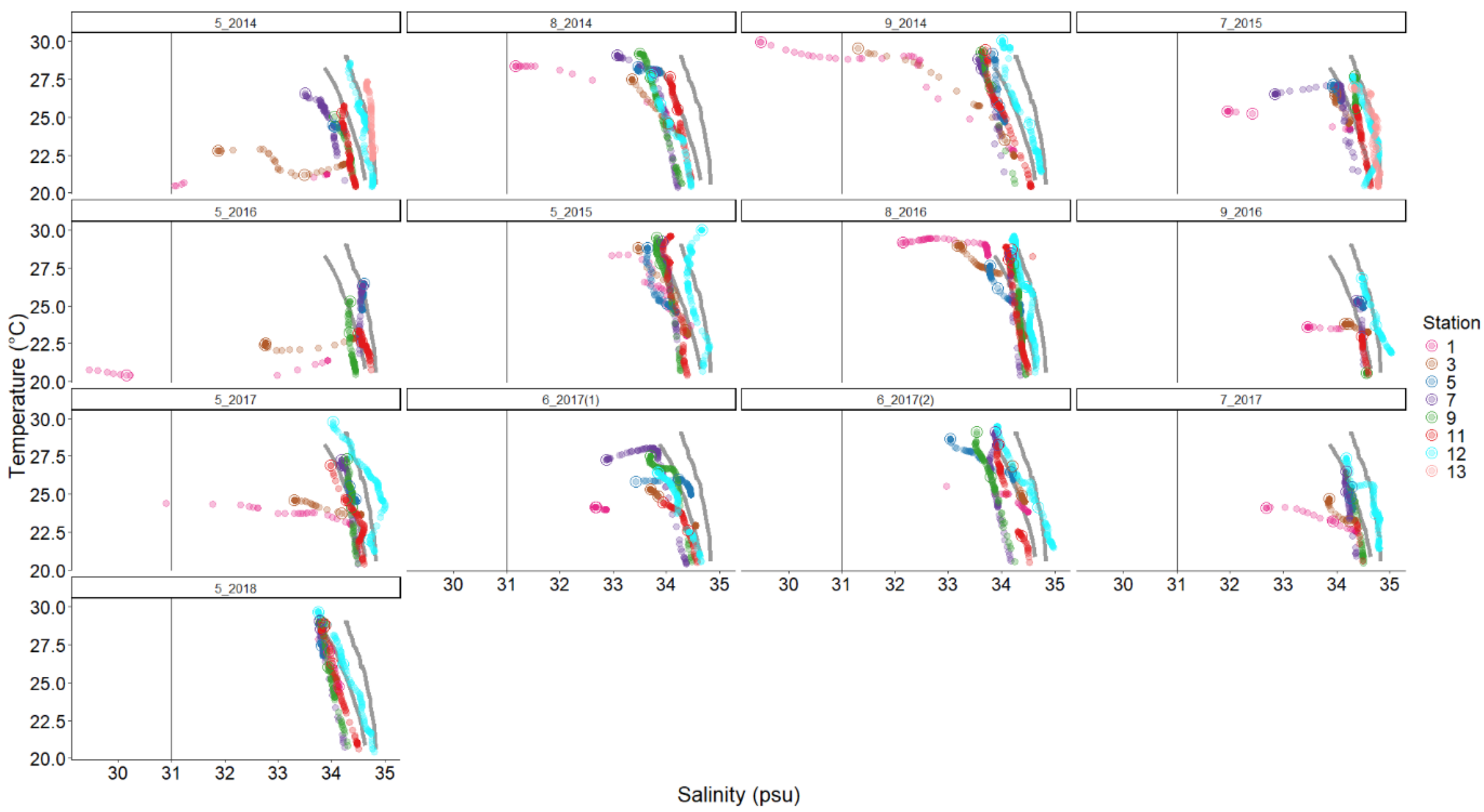

Figure S8. T-S diagrams of each sampling, in comparison with characteristic features of different water masses in the southern East China Sea, including the Kuroshio Current (KC), the China Coastal Current (CCC), and the Taiwan Warm Current (TWC). The area between two solid curves represents the KC, showing the average summer (June-August) T-S characteristics; the characteristic T-S of KC is determined based on historical observations at (N25°, E122.5°) and (N25°, E123°). The CCC is represented by the region to the left of the black vertical line (salinity: 31 psu). The TWC lies between the KC and CCC regions. The historical data was downloaded from the Ocean Data Bank, National Science and Technology Council (https://www.odb.ntu.edu.tw/).

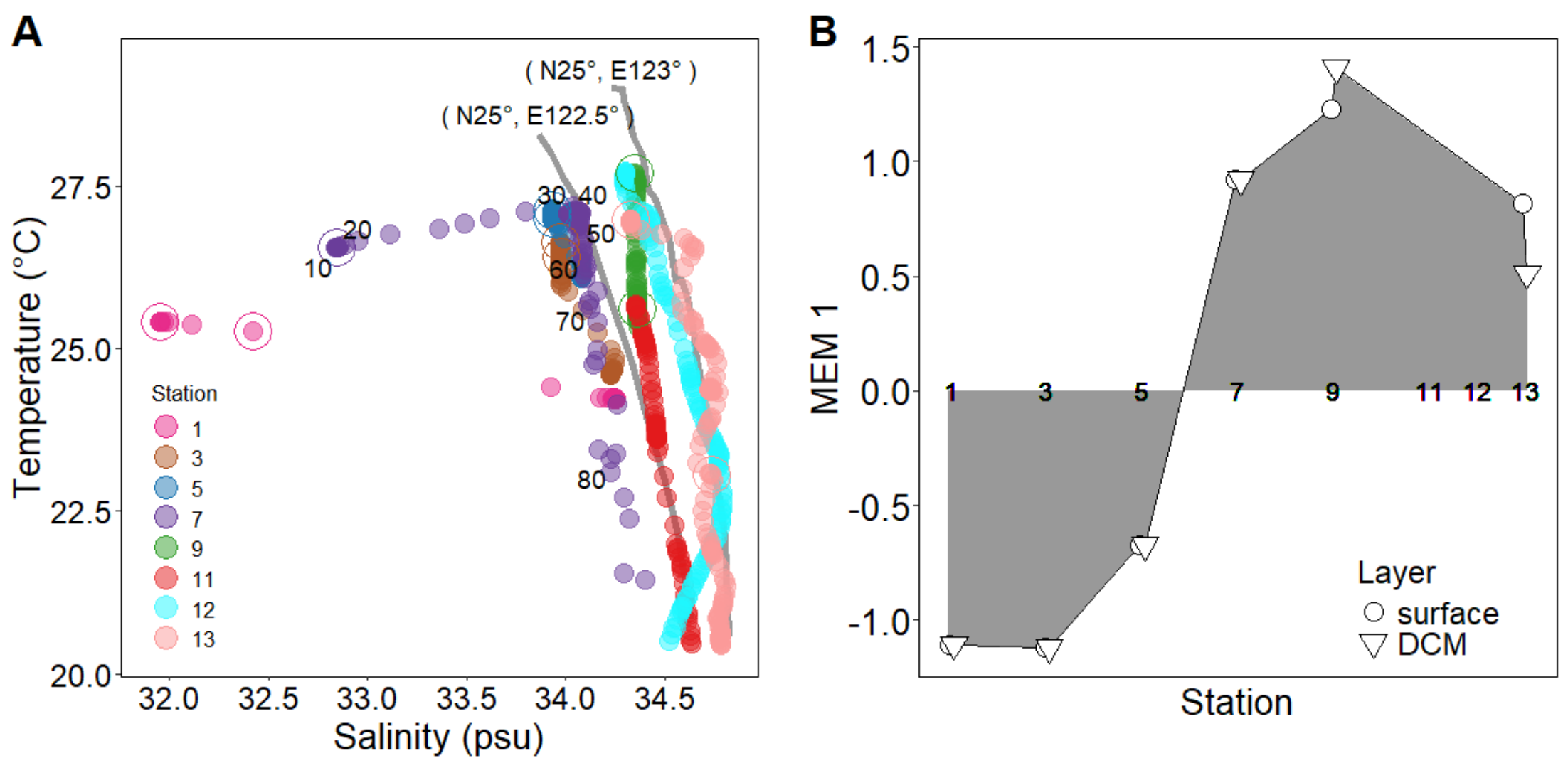

Figure S9. The dispersal limitation in cruise 7_2015. (A) The T-S diagram of each sampling, in comparison with characteristic features of different water masses in the southern East China Sea, including the Kuroshio Current (KC) and the Taiwan Warm Current (TWC). The area between two solid curves represents the KC, showing the average summer (June-August) T-S characteristics based on historical observations at (N25°, E122.5°) and (N25°, E123°). The TWC is represented by the region to the left of the KC area. The historical data was downloaded from the Ocean Data Bank, National Science and Technology Council (https://www.odb.ntu.edu.tw/). The hollow circle represents the sampling depth, and the numbers represent the depths of data points in station 7. (B) MEM 1 site scores across the transect. Circle and triangle represent surface and deep chlorophyll maximum (DCM) layers, respectively.

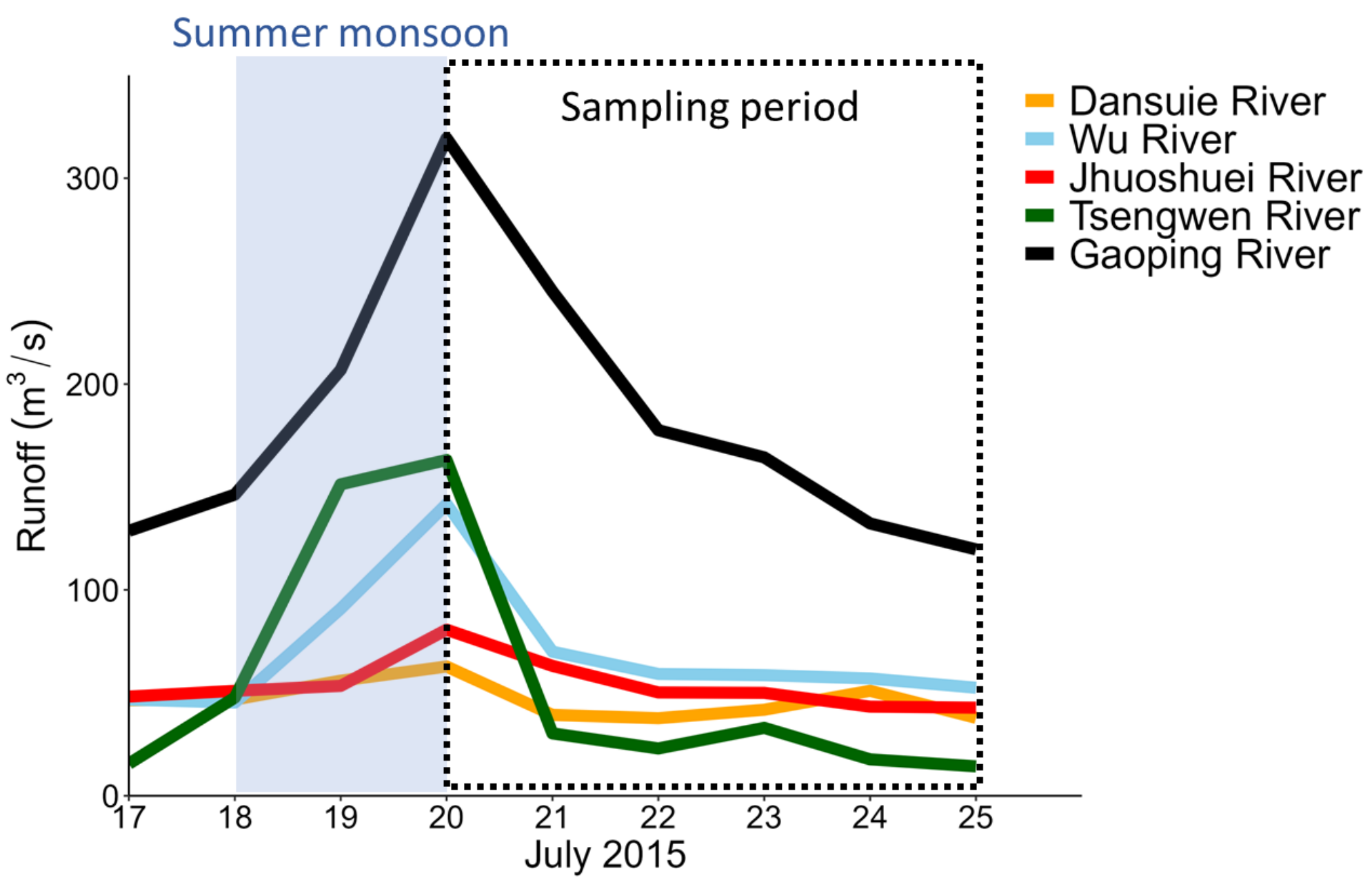

Figure S10. The runoff of main rivers during cruise 7_2015. Before the sampling period of the cruise (July 20–24, 2015), a rainfall event caused by the summer monsoon (July 18–19, 2015) revealed its influence on river runoff. The runoff data were downloaded from the Water Resources Agency, Ministry of Economic Affairs (https://gweb.wra.gov.tw/).
